# Association Between Fatty Liver Index and Incidence of Cataract Surgery in Individuals Aged 50 Years and Older Based on the Korean National Health Insurance Service-Health Screening Cohort (NHIS-HEALS) Data: Longitudinal Retrospective Cohort Study

**DOI:** 10.2196/57168

**Published:** 2024-11-14

**Authors:** Yonghwan Kim, Jeongsook Kim, Eoi Jong Seo, Kyung Tae Kim, Jae-woo Lee, Joungyoun Kim, Hee-Taik Kang

**Affiliations:** 1 Department of Family Medicine, CHA Bundang Medical Center, CHA University, Seongnam-si, Republic of Korea; 2 Clinical Research Team Hyundai Pharm Co., Seoul, Republic of Korea; 3 Department of Ophthalmology, College of Medicine, Chungbuk National University Hospital, Chungbuk National University, Cheongju-si, Republic of Korea; 4 Department of Family Medicine, Chungbuk National University Hospital, Cheongju, Republic of Korea; 5 Department of Family Medicine, Chungbuk National University College of Medicine, Cheongju, Chungbuk, Republic of Korea; 6 Department of Artificial Intelligence, University of Seoul, Seoul, Republic of Korea; 7 Department of Family Medicine, Severance Hospital, Yonsei University College of Medicine, 50-1 Yonsei-ro, Seodaemun-gu, Seoul, 03722, Republic of Korea, 82 2-2228-2330, 82 2-362-2330

**Keywords:** fatty liver index, nonalcoholic fatty liver disease, all-cataract, senile-cataract, surgery, NAFLD, Korean National Health Insurance Service, Health Screening Cohort, NHIS-HEALS

## Abstract

**Background:**

Cataract is a leading cause of vision impairment. Obesity-related risk factors, including insulin resistance, increase the risk of cataract. The fatty liver index (FLI) is a biomarker for noninvasive fat layer prediction of nonalcoholic fatty liver disease. The FLI has been used to evaluate the metabolic contribution in other organs besides the eye. However, no study exists on the FLI and eye disease.

**Objective:**

This retrospective cohort study for the association between the FLI and incidence of cataract surgery in individuals older than 50 years was designed to show that a higher FLI is associated with an increased incidence of cataract surgery in individuals aged 50 years and older.

**Methods:**

This study was retrospectively designed based on the Korean National Health Insurance Service-Health Screening Cohort (NHIS-HEALS) cohort (median follow-up of 9.8 years). Participants were assigned to 1 of 3 groups based on the FLI: low (FLI<30), intermediate (FLI 30-59), or high (FLI≥60). Kaplan-Meier survival analysis was performed on the cumulative incidence of all-cataract and senile-cataract surgery. Multivariable Cox proportional hazards regression models were used to study the association between the FLI group and cataract surgery after adjusting for potential confounders.

**Results:**

Of the 138,347 included participants, the incidence of cataract surgery was 12.49% (4779/38,274), 13.95% (6680/47,875), and 14.16% (7496/52,930) in the low, intermediate, and high FLI groups, respectively. After adjusting for all confounding factors, hazard ratios (HRs; 95% CIs) in the high FLI group for all-cataract surgery were 1.111 (1.028‐1.199) and 1.184 (1.101‐1.274) in men and women, respectively, when compared with the low FLI group. HRs (95% CIs) in the high FLI group for senile-cataract surgery were 1.106 (1.022‐1.197) and 1.147 (1.065‐1.237) in men and women, respectively, when compared with the low FLI group. The project was conducted between August 2023 and February 2024 without donations from external bodies.

**Conclusions:**

Individuals with a higher FLI had a higher risk of all-cataract surgery. This association was maintained even after limiting the analyses to senile-cataract surgery.

## Introduction

Cataract is a medical condition in which the normal clear lens of the eye or its capsule (the surrounding transparent membrane) becomes cloudy or opaque, impeding light from passing through to the retina [1]. If cataract is left untreated, the lens will continue to worsen, eventually resulting in loss of vision [2]. According to the World Health Organization, cataract is the leading cause of blindness among eye diseases, accounting for 47% of cases [3]. Cataract incidence increases with age, and risk factors include cigarette smoking, alcohol intake, diabetes, metabolic syndrome, and obesity [4]. Surgery is typically recommended when the cataract starts to interfere with a person’s daily activities or affects quality of life. In the early stages, cataract progression can be slowed using medications or eye drops such as iodide and pirenoxine [5,6], but the most definitive treatment method is surgery. Surgery is performed if poor vision causes discomfort in daily life or there is a risk of complications such as glaucoma [7,8].

Among the various risk factors associated with cataracts, obesity and obesity-related factors, such as diabetes and metabolic syndrome, are determinants. The association of obesity and fatty liver with cataract development has been reported in systematic reviews and meta-analyses. Obesity and fatty liver disease are complex metabolic conditions that can predispose individuals to cataract development through multiple interconnected pathways, including oxidative stress, inflammation, dyslipidemia, and vascular dysfunction [9,10].

Fatty liver disease, characterized by excessive fat accumulation in liver cells, is one of the most prevalent liver disorders worldwide [11]. Fatty liver may progress from simple steatosis to steatohepatitis, cirrhosis, and hepatocellular carcinoma [12]. Fatty liver is intimately associated with metabolic syndrome, type 2 diabetes, and cardiovascular disease [13].

The fatty liver index (FLI) was developed as a noninvasive marker of fatty liver. This index was created by matching various clinical imaging characteristics with formulas incorporating specific biochemical test results to estimate an individual’s risk of fatty liver disease [14]. Encompassing various factors associated with obesity, insulin resistance, and dyslipidemia, the FLI is an integrative risk assessment tool for fatty liver disease [15]. The FLI has been used to evaluate the metabolic contribution in other organs besides the eye. The positive association between the FLI and chronic kidney disease incidence can be elucidated through the comprehensive consideration of metabolic risk factors [16]. The FLI is associated with alterations in the blood coagulation system, potentially elevating the risk of thrombotic events, which may be influenced by metabolic factors [17]. However, no study exists on the FLI and eye disease to date.

We hypothesized that the degree of fatty liver would affect the meaningful incidence of cataracts requiring surgery. Therefore, in this study, we aimed to investigate the incidence of cataract surgery according to the interval of the FLI.

## Methods

### Data Source

The National Health Insurance Service-Health Screening Cohort (NHIS-HEALS) is a cohort of participants who participated in health-screening programs provided by the NHIS in the Republic of Korea. The NHIS created the NHIS-HEALS cohort database in 2019 to offer relevant and useful data to health researchers, particularly those in the field of noncommunicable diseases and health risk factors, as well as policy makers [18,19]. The Korean NHIS-HEALS database contains sociodemographic data, laboratory data from health checkups, surgery codes, disease codes, and the cause of death, among other variables. The database also contains information about social lifestyle behaviors such as cigarette smoking, alcohol consumption, and physical activity.

### Participants

We used data from the Korean NHIS-HEALS database, which represents 514,794 individuals randomly sampled from 5.1 million Koreans included in the health examination database. All individuals in this sampled cohort underwent a national health checkup between 2009 and 2010 and were between 50 and 79 years of age in December 2009. Among this sampled cohort, our study focused on those diagnosed with cataract surgery since 2009.

### Follow-Up Investigation

**Figure 1.**
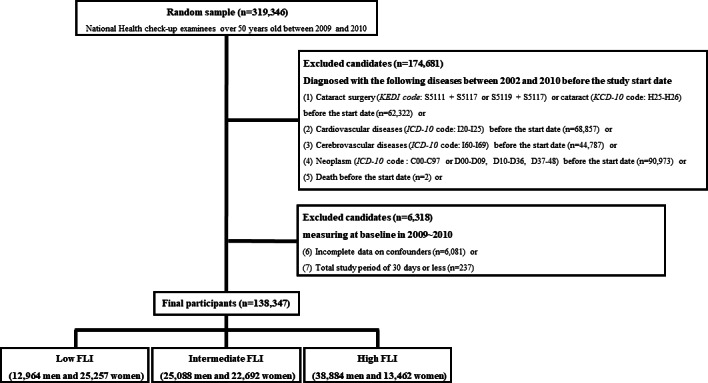
Flowchart of the study. FLI: fatty liver index; *ICD-10*: *International Classification of Diseases, Tenth Revision*; *KCD-10*: *Korean Standard Classification of Diseases, Tenth Revision*; KEDI: Korean Electronic Data Interchange.

The flowchart in Figure 1 describes how individuals were selected from the NHIS-HEALS database using the inclusion and exclusion criteria for this study. Initially, we selected 319,346 individuals aged 50 years or older between 2009 and 2010 who underwent a national health checkup. Among them, 174,681 individuals were excluded for the following reasons: (1) diagnosed with a cataract (*Korean Standard Classification of Diseases, Eighth Revision* [*KCD-*8] codes: H25, H26) and underwent cataract surgery (Korean Electronic Data Interchange [KEDI] code S5111 + S5117 or S5119 + S5117) before the study start date (n=62,322); (2) diagnosed with cardiovascular diseases (*International Classification of Diseases, Tenth Revision* [*ICD-10*] codes: I20-I25) before the study start date (n=68,857); (3) diagnosed with cerebrovascular diseases (*ICD-10* codes: I60-I69) before the study start date (n=44,787); (4) diagnosed with neoplasms (*ICD-10* codes: C00-C97 or D00-09, D10-36, D37-48) before the study start date (n=90,973); (5) death between 2009 and 2010 (n=2); (6) incomplete data for confounders between 2009 and 2010 (n=6081); and (7) a total study duration of ≤30 days between 2009 and 2010 (n=237). Therefore, we included 138,347 participants (76,936 men and 61,411 women) in our analyses. Participants were excluded according to the abovementioned criteria, but the values corresponding to each exclusion criterion were not mutually exclusive.

### Study Variables and Measurements

#### Fatty Liver Index

The FLI is a biomarker for noninvasive fat layer prediction of nonalcoholic fatty liver disease (NAFLD). It has been validated against imaging methods for fatty liver diagnosis in the United States, with an accuracy of 0.84 (95% CI 0.81-0.87) [14]. Also, the FLI was validated with an area under the curve value of 0.87 or 0.785 in the Korean population [20,21]. Participants were classified into 1 of 3 groups based on the FLI: low risk (FLI<30), intermediate risk (FLI 30‐59), or high risk (FLI≥60). The FLI was calculated using the following formula represented in [14].

#### Outcomes and Study Duration

The primary outcome of this study was cataract surgery rather than cataract to discriminate clinically significant cataract from overall cataract. In the early stages of cataract, most patients do not visit the hospital and often do not even realize that they are affected. Cataract surgery was defined as a *KEDI* code that concurrently claimed “extracapsular or intracapsular extraction” and “primary intraocular lens implantation” (*KEDI* codes S5111 and S5117, respectively) or “phacoemulsification” and “primary intraocular lens implantation” (*KEDI* codes S5119 and S5117) on the same day. Eventually, as an operational definition, cataract surgery was defined as the primary outcome for the abovementioned cataract surgery including *KCD* codes of age-related cataract (*KCD* H25) and other cataract (*KCD* H26). Senile-cataract surgery was defined as the secondary outcome for those who experienced the abovementioned cataract surgery including *KCD* codes of age-related cataract (*KCD* H25). The beginning date of the study was the date of the first health examination between 2009 and 2010. The end of the study for each individual was the date of cataract surgery or death after the start of the study. The available data allowed for the analysis to be conducted only until the end of 2019. If neither happened, the end date was the earlier date of the last outpatient visit or the last medical health checkup.

#### Confounding Variables

We considered age, smoking status, alcohol consumption, physical activity, household income, BMI, systolic blood pressure (SBP), total serum cholesterol, preoperative ocular characteristics, hypertension, diabetes mellitus, and dyslipidemia at the baseline health checkup as confounding variables.

BMI (kg/m^2^) was calculated as body weight (kg) divided by height squared (m^2^). Participants were classified as nonsmokers (individuals who never smoked or had ever smoked less than 100 cigarettes in the past), former smokers (individuals who had ever smoked but did not currently), and current smokers (individuals who currently smoked and had ever smoked 100 cigarettes or more). Alcohol consumption was categorized as rare (less than 2 drinks per month), moderate (3 drinks per month to 2 drinks per week), or heavy (3 or more drinks per week). Physical activity was divided into 3 categories: low (not meeting the definition of “moderate” nor “high”), moderate (5 or more days of moderate-intensity exercises per week; 5 or more days of a combination of moderate-intensity or vigorous-intensity exercises per week), and high (3 or more days of vigorous-intensity exercises per week; 7 or more days of a combination of moderate-intensity or vigorous-intensity exercises per week). Economic status was grouped based on the self-reported monthly household income as low (<30th percentile), middle (30th-69th percentile), or high (70th-100th percentile).

Preoperative ocular characteristics were defined by the diagnostic *KCD* codes of hyperopia (*KCD* H52.0), myopia (*KCD* H52.1, H44.2), history of eye trauma (*KCD* S05), and history of retinal detachment (*KCD* H33). Because possible effects from preoperative ocular characteristics on the association between the FLI and cataract may exist, we further looked into how eye trauma, vitrectomy, uveitis, scleritis, and the use of topical steroids could effect the association between the FLI and cataract surgery.

Hypertension was defined based on a disease diagnosis code of hypertension (I10-I15) and any prescription for antihypertensives (angiotensin-converting enzyme inhibitors, angiotensin receptor blockers, calcium channel blockers, β-blockers, α-blockers, diuretics, other vasodilators, and combinations). Dyslipidemia was defined as a dyslipidemia diagnosis code (E78.0-E78.9) and any prescription of antidyslipidemic drugs (statins, fibrates, omega-3 fatty acids, niacin, or bile acid sequestrants) for ≥90 days up to the date of inclusion.

Diabetes mellitus was defined as (1) fasting glucose ≥126 mg/dL, or (2) a diagnosis code of diabetes mellitus (E10-E14) and antidiabetics (metformin, sulfonylurea, thiazolidinedione, inhibitor of dipeptidyl peptidase 4, sodium-glucose cotransporter 2 inhibitors, insulin, glucagon-like peptide 1 receptor agonists, and combinations) for 90 days or longer up to the date of inclusion.

### Statistical Analysis

#### Baseline Characteristics

No statistical power calculation was conducted before the study because this study was retrospectively designed using the Korean NHIS-HEALS cohort. Continuous variables are presented as mean (SD). Categorical variables are expressed as the number of participants (percentage). ANOVA was conducted for continuous variables and chi-square testing for categorical variables to assess the significance of differences between groups.

#### Survival Analysis

Kaplan-Meier estimates and the log-rank test were used to compare survival outcomes. To determine the effects of confounding factors on the primary composite outcome, 3 Cox proportional hazards regression models were used: model 1 was adjusted for age; model 2 was adjusted for age, smoking status, alcohol consumption, physical activity, and economic status; and model 3 was adjusted for model 2 plus BMI, SBP, total serum cholesterol, preoperative ocular characteristics, hypertension, diabetes mellitus, and dyslipidemia. All *P* values were 2-sided, and the significance level was .05.

#### Software

Statistical analyses were executed using SAS Enterprise Guide 7.1 software (SAS Inc) and R Studio version 3.3.3 (The R Foundation).

### Ethical Considerations

The institutional review board of Chungbuk National University Hospital approved this study (CBNUH 2023-08-035), which was conducted in accordance with the Declaration of Helsinki (1975). All Korean NHIS-HEALS data are provided anonymously. Informed consent was waived for Korean NHIS-HEALS data by the ethics committee of the Korean NHIS service because the data were fully anonymized at all stages, including data cleaning and statistical analysis. Because the research was conducted using health insurance claims data, compensation provided to human research participants was not applicable.

## Results

### Characteristics of Study Participants

The 138,347 participants were divided into low (12,964 men and 25,257 women), intermediate (25,088 men and 22,692 women), and high (38,884 men and 13,462 women) FLI groups. The median follow-up duration was 9.8 years. The primary composite outcome (all-cataract surgery) occurred in 12.49% (4774/38,221), 13.96% (6670/47,780), and 14.19% (7428/52,346) of the low, intermediate, and high FLI groups, respectively.

Table 1 shows the baseline characteristics of the study population by sex. Mean age of the low, intermediate, and high FLI groups was 58.60, 57.57, and 56.72 years for men, respectively, and 56.31, 58.16, and 59.09 years for women. In both sexes, the high FLI group had the highest BMI, SBP, fasting blood glucose, and total cholesterol levels. Individuals in the high FLI group were more likely to be heavy alcohol drinkers and have comorbid conditions such as hypertension, dyslipidemia, and diabetes mellitus.

**Table 1.** Baseline characteristics of the study population by sex.

	Men				Women			
	Low FLI^a^	Intermediate FLI	High FLI	*P* value^b^	Low FLI	Intermediate FLI	High FLI	*P* value^b^
Number	12,964	25,088	38,884	N/A^c^	25,257	22,692	13,462	N/A
Age (years), mean (SD)	58.60 (7.91)	57.57 (7.10)	56.72 (6.43)	<.001	56.31 (6.74)	58.16 (7.13)	59.09 (7.17)	<.001
BMI (kg/m^2^), mean (SD)	20.85 (1.90)	23.14 (1.83)	25.52 (2.38)	<.001	21.74 (1.95)	24.25 (1.98)	27.06 (2.79)	<.001
SBP^d^ (mm Hg), mean (SD)	121.13 (14.61)	124.94 (14.43)	129.22 (14.62)	<.001	118.90 (14.64)	124.25 (15.23)	128.78 (15.62)	<.001
FBG^e^ (mg/dL), mean (SD)	95.96 (20.01)	99.65 (23.57)	106.98 (29.63)	<.001	92.88 (15.35)	97.04 (19.93)	103.68 (26.46)	<.001
Total cholesterol (mg/dL), mean (SD)	184.17 (31.17)	195.30 (33.55)	204.55 (37.41)	<.001	201.11 (34.59)	211.21 (37.07)	219.02 (39.92)	<.001
**Smoking status, n (%)**				<.001				<.001
Never smoker	5313 (41.0)	9332 (37.2)	12,282 (31.6)		24,751 (98.0)	22,203 (97.8)	13,077 (97.2)	
Former smoker	3442 (26.5)	7827 (31.2)	12,733 (32.7)		155 (0.6)	157 (0.7)	100 (0.7)	
Current smoker	4209 (32.5)	7929 (31.6)	13,869 (35.7)		352 (1.4)	332 (1.5)	285 (2.1)	
**Drinking status, n (%)**				<.001				<.001
Rare	5983 (46.2)	9771 (38.9)	11,371 (29.2)		21,469 (85.0)	19,403 (85.5)	11,384 (84.6)	
Moderate	4781 (36.9)	10,060 (40.1)	15,738 (40.5)		3215 (12.7)	2741 (12.1)	1663 (12.3)	
Heavy	2200 (17.0)	5257 (21.0)	11,775 (30.3)		577 (2.3)	548 (2.4)	415 (3.1)	
**Physical activity, n (%)**				<.001				<.001
Low	8676 (66.9)	16,111 (64.2)	25,338 (65.3)		18,147 (71.8)	16,909 (74.5)	10,415 (77.4)	
Moderate	1225 (9.4)	2609 (10.4)	4283 (11.0)		2048 (8.1)	1675 (7.4)	873 (6.5)	
High	3063 (23.6)	6368 (25.4)	9213 (23.7)		5062 (20.0)	4108 (18.1)	2174 (16.1)	
**Household income, n (%)**				<.001				<.001
Low	2617 (20.2)	4479 (17.9)	7053 (18.1)		6723 (26.6)	5896 (26.0)	3654 (27.2)	
Middle	3186 (24.6)	5665 (22.6)	8540 (22.0)		6478 (25.6)	5967 (26.3)	3728 (27.7)	
High	7161 (55.2)	14,944 (59.6)	23,291 (59.9)		12,056 (47.7)	10,829 (47.7)	6080 (45.2)	
Hypertension, n (%)	1503 (11.6)	4950 (19.7)	12,085 (31.1)	<.001	3423 (13.6)	5602 (24.7)	5164 (38.4)	<.001
Diabetes, n (%)	864 (6.7)	2724 (10.9)	7526 (19.4)	<.001	933 (3.7)	1777 (7.8)	2232 (16.6)	<.001
Dyslipidemia, n (%)	373 (2.9)	1637 (6.5)	5079 (13.1)	<.001	2013 (8.0)	3507 (15.5)	3134 (23.3)	<.001
Preoperative ocular disease^f^, n (%)	1829 (14.1)	3641 (13.4)	5226 (13.4)	<.001	4649 (18.4)	3774 (16.6)	2084 (15.5)	<.001

a FLI: fatty liver index.

b *P* values are determined by ANOVA for continuous variables and chi-square testing for categorical variables.

c N/A: not applicable.

d SBP: systolic blood pressure.

e FBG: fasting blood glucose.

f Preoperative ocular disease consisted of hyperopia, myopia, history of eye trauma, and history of retinal detachment.

### Cumulative Incidence Between FLI and All-Cataract Surgery in Men and Women

**Figure 2.**
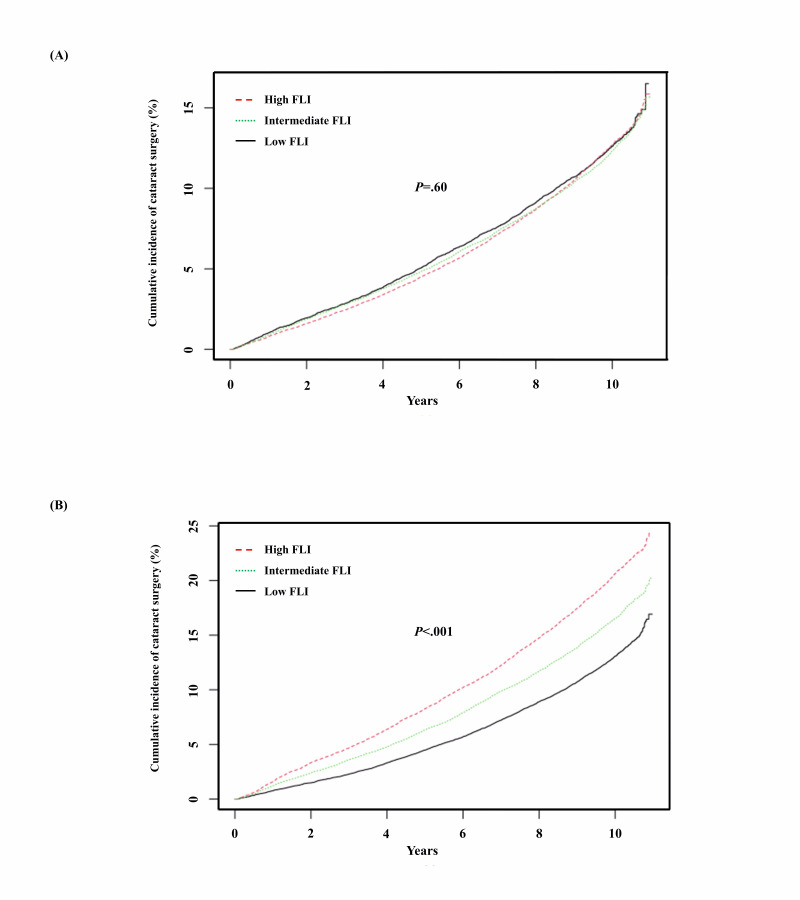
Cumulative incidence of all-cataract surgery according to FLI by sex. (A) Men and (B) women older than 50 years from Kaplan-Meier survival analysis. FLI: fatty liver index.

Figure 2A and B show the estimates for the cumulative incidence rates of all-cataract surgery, which are equal to 1 minus the Kaplan-Meier estimates. The estimated incidence of all-cataract surgery in women was highest in the high FLI group and lowest in the low FLI group (*P*<.001) (Figure 2B). However, there was no difference in the estimated incidence of cataract surgery among the 3 FLI groups in men (*P*=.600) (Figure 2A).

**Figure 3.**
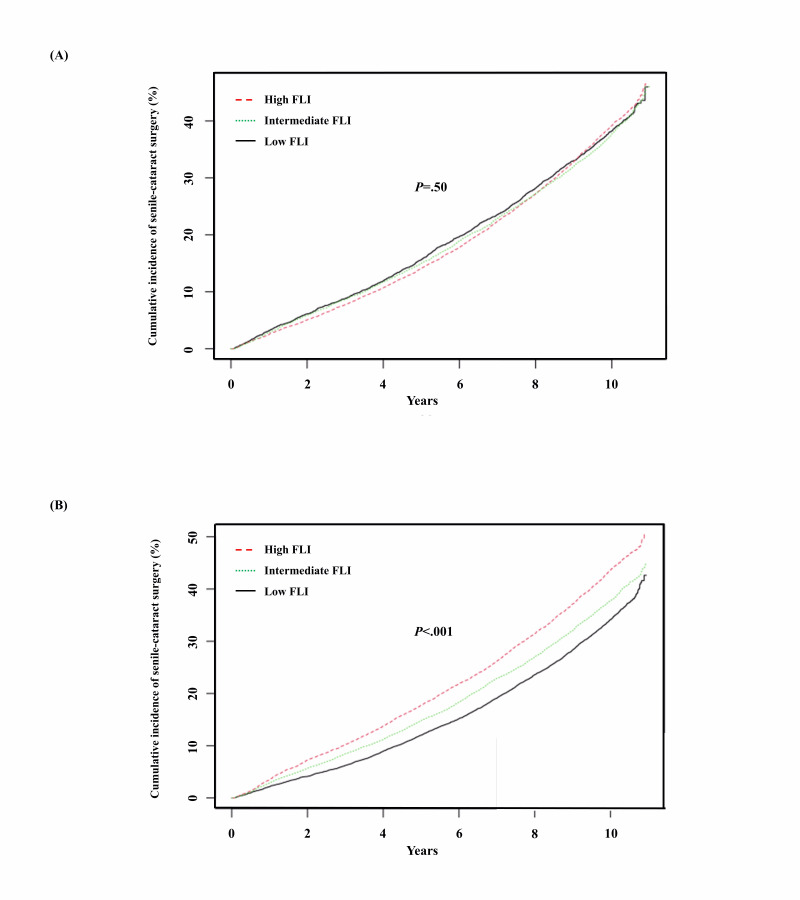
Cumulative incidence of senile-cataract surgery according to FLI by sex. (A) Men and (B) women older than 50 years from Kaplan-Meier survival analysis. FLI: fatty liver index.

Figure 3A and B show the estimates for the cumulative incidence rates of senile-cataract surgery, which are equal to 1 minus the Kaplan-Meier estimates. The estimated incidence of all-cataract surgery in women was highest in the high FLI group and lowest in the low FLI group (*P*<.001) (Figure 3B). However, there was no difference in the estimated incidence of cataract surgery among the 3 FLI groups in men (*P*=.500) (Figure 3A).

### Associations Between FLI and All-Cataract Surgery

Results of the Cox proportional hazards regression models to investigate the association between the FLI and the incidence of all-cataract surgery are shown in Table 2. Compared with the low FLI group, hazard ratios (HRs; 95% CIs) of the intermediate and high FLI groups for all-cataract surgery were 1.102 (1.036‐1.172) and 1.246 (1.175‐1.320), respectively, in men and 1.092 (1.042‐1.145) and 1.288 (1.224‐1.356) in women after adjusting for age only. Adjusted HRs (95% CIs) for the intermediate and high FLI groups were 1.050 (0.983‐1.123) and 1.111 (1.028‐1.199), respectively, in men and 1.054 (0.999‐1.111) and 1.184 (1.101‐1.274) in women compared with those for the low FLI group after adjustment for all confounding variables.

**Table 2.** Multivariable Cox proportional hazards regression models for all-cataract surgery by FLI^a^ interval over 50 years.

	Men			Women		
	Low FLI (reference)	Intermediate FLI, hazard ratio (95% CI)	High FLI, hazard ratio (95% CI)	Low FLI (reference)	Intermediate FLI, hazard ratio (95% CI)	High FLI, hazard ratio (95% CI)
Model 1^b^	1	1.102 (1.036‐1.172)^c^	1.246 (1.175‐1.320)^c^	1	1.092 (1.042‐1.145)^c^	1.288 (1.224‐1.356)^c^
Model 2^d^	1	1.095 (1.029‐1.164)^c^	1.223 (1.154‐1.297)^c^	1	1.092 (1.042‐1.145)^c^	1.289 (1.224‐1.357)^c^
Model 3^e^	1	1.050 (0.983-1.123)^f^	1.111 (1.028‐1.199)^c^	1	1.054 (0.999‐1.111)^g^	1.184 (1.101‐1.274)^c^

a FLI: fatty liver index.

b Model 1: adjusted for age.

c *P*<.001.

d Model 2: adjusted for smoking status, drinking status, physical activity, and economic status in addition to the variable of model 1.

e Model 3: adjusted for BMI, systolic blood pressure, total cholesterol, preoperative ocular characteristics, diabetes mellitus, dyslipidemia, and hypertension in addition to variables of model 2.

f *P*=.15.

g *P*=.06.

Because cataracts are highly correlated with age, we conducted further statistical analyses after separating senile-cataract surgery as a secondary outcome from all-cataract surgery. Table 3 shows the association between the FLI and senile-cataract surgery. Compared with the low FLI group, adjusted HRs (95% CIs) for the high FLI group were 1.106 (1.022-1.197) in men and 1.147 (1.065-1.237) in women after full adjustment, which is a significant difference. However, there was no association in the intermediate FLI group for either sex (HR 1.034, 95% CI 0.966-1.108 in men and HR 1.003, 95% CI 0.950-1.060 in women).

**Table 3.** Multivariable Cox proportional hazards regression models for senile-cataract surgery by FLI^a^ interval over 50 years.

	Men			Women		
	Low FLI (reference)	Intermediate FLI, hazard ratio (95% CI)	High FLI, hazard ratio (95% CI)	Low FLI (reference)	Intermediate FLI, hazard ratio (95% CI)	High FLI, hazard ratio (95% CI)
Model 1^b^	1	1.038 (0.975-1.107)^c^	1.130 (1.065‐1.200)^d^	1	1.012 (0.964‐1.062)^e^	1.180 (1.120‐1.244)^d^
Model 2^f^	1	1.034 (0.970‐1.101)^g^	1.109 (1.044‐1.178)^d^	1	1.011 (0.964‐1.062)^h^	1.177 (1.117‐1.241)^d^
Model 3^i^	1	1.034 (0.966–1.108)^j^	1.106 (1.022‐1.197)^d^	1	1.003 (0.950-1.060)^k^	1.147 (1.065‐1.237)^d^

a FLI: fatty liver index.

b Model 1: adjusted for age.

c *P*=.24.

d *P*<.05.

e *P*=.63.

f Model 2: adjusted for smoking status, drinking status, physical activity, and economic status in addition to the variable of model 1.

g *P*=.31.

h *P*=.65.

i Model 3: adjusted for BMI, systolic blood pressure, total cholesterol, preoperative ocular characteristics, diabetes mellitus, dyslipidemia, and hypertension in addition to variables of model 2.

j *P*=.34.

k *P*=.91.

### Analysis for the Common Cause of the Non–Age-Related Cataract

Additional analyses were performed to investigate common causes of non–age-related cataracts, specifically to exclude the effects of factors such as trauma, vitrectomy, uveitis, scleritis, and the use of topical steroids (Multimedia Appendix 2).

Among these factors, only steroid use (including topical, intravenous, oral, and inhaler steroids) was shown to be statistically significant. Compared with the low FLI group, HRs (95% CIs) of the intermediate and high FLI groups for the steroid use were 1.035 (0.968‐1.107) and 1.099 (1.017‐1.188), respectively, in men and 1.049 (0.994‐1.106) and 1.179 (1.096‐1.269), respectively, in women after full adjustment (Table 4).

**Table 4.** Multivariable Cox proportional hazards regression models for all-cataract surgery by FLI^a^ interval over 50 years according to the use of steroids (sum of topical, intravenous, oral, and inhaler steroids).^b^

Steroid	Sex	Low FLI (reference)	Intermediate FLI, hazard ratio (95% CI)	High FLI, hazard ratio (95% CI)
Yes	Men	1	1.035 (0.968‐1.107)	1.099 (1.017‐1.188)
Yes	Women	1	1.049 (0.994‐1.106)	1.179 (1.096‐1.269)
No	Men	1	1.486 (0.950‐2.324)	1.168 (0.679‐2.010)
No	Women	1	0.937 (0.459‐1.913)	1.128 (0.427‐2.984)

a FLI: fatty liver index.

b Adjusted for age, smoking status, drinking status, physical activity, economic status, BMI, SBP, total cholesterol, preoperative ocular characteristics, diabetes mellitus, dyslipidemia, and hypertension.

## Discussion

### Principal Findings

This retrospective cohort study found that individuals with higher FLI scores, indicative of definite fatty liver, show a higher incidence of cataract surgery based on data from the Korean NHIS-HEALS database with comprehensive adjustment for potential confounders. These findings provide evidence that higher fatty liver, as estimated by the FLI, induced more cataracts severe enough to warrant surgical intervention.

A previous meta-analysis showed longitudinal associations of obesity with incident age-related cataract [9]. Our study focused on the potential association between fatty liver, an obesity-related condition, and cataracts. Several potential mechanisms could underlie the relationship between obesity or fatty liver and cataracts. Specifically, oxidative stress, inflammation, and insulin resistance, all of which are characteristic of obesity, may affect the lens of the eye and lead to cataract formation [22-28].

Oxidative stress plays an important role in the onset and progression of cataracts. The FLI is a reliable indicator of hepatic steatosis and could reflect the impact of oxidative stress on the lens of the eye. Glutathione peroxidase is an enzyme that plays a crucial role in removing lipid hydroperoxides, protecting cell membranes from oxidative damage. With aging, there is a decrease in the antioxidant properties of the lens, evidenced by a reduction in substances such as glutathione and the expression of antioxidant products such as malondialdehyde (MDA). MDA is a significant end product of free radical reactions on membrane fatty acids and is a marker of oxidative stress [22]. Kaur et al [23] reported that MDA level was increased in patients with cataract. This suggests that oxidative stress may contribute to the development of cataracts. Similarly, oxidative stress has been implicated in the pathogenesis of NAFLD. An increase in oxidative stress can lead to liver damage and fat accumulation, which is quantitatively assessed using the FLI [24]. Therefore, cataract and NAFLD have a common underlying mechanism related to oxidative stress and lipid peroxidation.

Adipose tissue releases various inflammatory mediators, such as interleukin (IL)–6 and IL-8 [25]. Chronic systemic inflammation has been implicated in various diseases such as NAFLD, and it is possible that these mediators affect the physiology of the eye, contributing indirectly to uveitis and cataract development [26].

Insulin resistance is a key feature of NAFLD and is important in the pathogenesis and progression of NAFLD to nonalcoholic steatohepatitis [29,30]. Several papers have reported a correlation between insulin resistance and cataract. Hyperglycemia results in the formation of advanced glycation end products, which can modify lens proteins, leading to cross-linking, aggregation, and clouding [22]. Maralani and colleagues [27] demonstrated that high blood glucose and obesity predicted a 5-year increase in the incidence of cortical cataract, while low high-density lipoprotein and high glucose were associated with an increased 10-year incidence of cortical and posterior subcapsular cataracts. Chang et al [28] reported that metabolic syndrome was significantly associated with cataracts (odds ratio 1.129, 95% CI 1.0175‐1.184). As metabolic syndrome, high blood sugar, and obesity are strongly related to insulin resistance [31-33], insulin resistance in NAFLD is likely to be associated with cataracts.

Our study had several limitations. First, the FLI is an indirect measure of fatty liver, and direct imaging or biopsy data were not available for our Korean NHIS-HEALS cohort. However, imaging studies and biopsies are expensive and may involve complications. The FLI is a good alternative to imaging or biopsy studies. Second, the NHIS-HEALS cohort database was constructed to gather information to reimburse insurance claims. Because these retrospective cohort data were not collected for research purposes, clinical information about cataract severity, difficulty of cataract surgery, and side effects were not recorded. However, to minimize overdiagnosis of cataracts and account for differences in cataract severity, we used cataract surgery as our primary outcome. Third, selection bias may have been present as the decision to perform cataract surgery could differ according to the ophthalmologist’s experiences and beliefs. In addition, whether surgery is performed may vary depending on insurance reimbursement coverage standards. Fourth, because the amount of outdoor and indoor work or activities may vary among individuals, UV light could cause different results for the prevalence of cataracts. However, our Korean NHIS-HEALS data do not include information on UV lights. Therefore, we could not report the effect of UV light. Fifth, cataract is categorized into age-related or non–age related. Although we adjusted for the preoperative ocular characteristics, common causes of non–age-related cataract such as trauma, vitrectomy, uveitis, scleritis, and use of topical steroid could effect the association between the FLI and cataract surgery. In our additional analysis of non–age-related factors, use of steroid contributes to higher risk of cataract in people with a higher FLI. It seems that steroid use may influence the association between the FLI and cataract surgery.

Strengths of this study include its large-scale nature with long-term follow-up data (2009‐2018) for adults older than 50 years. The use of cataract surgery, which indicates severe cataract, as the main outcome is a unique strength. The NHIS-HEALS cohort database contains national insurance claims data collected during routine health checkups of a representative sample of the Korean population older than 40 years who are automatically mandatorily registered. Therefore, this insurance system data may have little selection bias.

We have performed separate survival analyses for men and women to account for potential sex-specific differences in the relationship between the FLI and the incidence of cataract surgery, allowing for more nuanced interpretations of the results. Based on an additional analysis of the unadjusted model to exclude any interference effect due to age for justifying survival analysis (Multimedia Appendix 3), age is suggested to be a crucial factor influencing the risk of cataracts in men and must be controlled to reveal the underlying relationship with the FLI. Understanding these sex-specific differences is crucial to developing targeted prevention and intervention strategies. The significant impact of age on this association in men suggests that age-specific measures could be more critical for this group. Meanwhile, for women, direct management of fatty liver disease might play a more central role in reducing the risk of cataracts.

Nonetheless, a subgroup analysis of those younger than 60 years and those older than 60 years (Multimedia Appendix 4) showed that women older than 60 years were statistically associated with cataract surgery in the intermediate and advanced FLI groups. This fact means that fatty liver is an important factor in the occurrence of cataract surgery even in this age group.

Furthermore, because the FLI can also be assessed using outpatient treatment, our study findings have a broader applicability. Finally, this study is the first in which an association between a higher FLI and higher incidence of cataract surgery was reported.

### Conclusions

Our findings indicate an association between high risk of fatty liver disease, as estimated by the FLI, and increased frequency of cataract surgery based on data from the NHIS-HEALS cohort. The association between a higher FLI score and cataract surgery was more prominent in women than in men.

Further research is needed to elucidate the mechanisms underlying the association between fatty liver and cataract and to explore potential therapeutic interventions that might mitigate this association.

## Supplementary material

10.2196/57168 Multimedia Appendix 1 The equation of fatty liver index.

10.2196/57168 Multimedia Appendix 2 Additional analysis for the common cause of non–age-related cataracts.

10.2196/57168 Multimedia Appendix 3 Additional unadjusted model for Tables2  3.

10.2196/57168 Multimedia Appendix 4 Additional analysis for age before and after the age of 60 years.

## Abbreviations

FLI: fatty liver index
HR: hazard ratio
*ICD-10*: *International Classification of Diseases, Tenth Revision*
IL: interleukin
*KCD-8*: *Korean Standard Classification of Diseases, Eighth Revision*
KEDI: Korean Electronic Data Interchange
MDA: malondialdehyde
NAFLD: nonalcoholic fatty liver disease
NHIS-HEALS: National Health Insurance Service-Health Screening Cohort
SBP: systolic blood pressure
