# Supplementary material for: Association Between Fatty Liver Index and Incidence of Cataract Surgery in Individuals Aged 50 Years and Older Based on the Korean National Health Insurance Service-Health Screening Cohort (NHIS-HEALS) Data: Longitudinal Retrospective Cohort Study
Source: JMIR Public Health Surveill. 2024 Nov 14;10:e57168. doi: 10.2196/57168 (PMC11581417; doi:10.2196/57168)
Supplement: Multimedia Appendix 1 [file publichealth-v10-e57168-s001.docx]

Appendix 1.

**Fatty Liver Index Equation**

FLI was calculated using the following formula:

FLI= $\frac{1}{[1+exp(-x)]} \times100$

$x$ = 0.953 $\times$ $\log_{e} \left( serum triglycerides \right)+0.139 \times(body mass index [BMI]$

+ 0.718 $\times$ $\log_{e} \left( serum \gamma-glutamyl transpeptidase \right)$

+ 0.053 $\times$ ($waist circumference$) – 15.745

**References**

1. Bedogni G, Bellentani S, Miglioli L, et al. The Fatty Liver Index: a simple and accurate predictor of hepatic steatosis in the general population. *BMC Gastroenterol*. 2006;6:33. <https://pubmed.ncbi.nlm.nih.gov/17081293/>
