# Supplementary material for: Association Between Fatty Liver Index and Incidence of Cataract Surgery in Individuals Aged 50 Years and Older Based on the Korean National Health Insurance Service-Health Screening Cohort (NHIS-HEALS) Data: Longitudinal Retrospective Cohort Study
Source: JMIR Public Health Surveill. 2024 Nov 14;10:e57168. doi: 10.2196/57168 (PMC11581417; doi:10.2196/57168)
Supplement: Multimedia Appendix 2 [file publichealth-v10-e57168-s002.docx]

Appendix 2.

**A**. Multivariable Cox proportional hazards regression models for history of eye trauma (KEDI S05)

| **Eye Trauma** | Sex | Low FLI | Intermediate FLI | High FLI |
| --- | --- | --- | --- | --- |
| Yes | Men | 1 | 1.123 (0.815–1.549) | 0.966 (0.664–1.406) |
|  | Women | 1 | 0.880 (0.596–1.300) | 0.944 (0.553–1.611) |
| No | Men | 1 | 1.049 (0.980–1.122) | 1.118 (1.033–1.209) |
|  | Women | 1 | 1.056 (1.000–1.114) | 1.186 (1.101–1.277) |

FLI: fatty liver index

Adjusted for age, smoking status, drinking status, physical activity, economic status, body mass index, systolic blood pressure, total cholesterol, preoperative ocular characteristics, diabetes mellitus, dyslipidaemia, and hypertension.

**B**. Multivariable Cox proportional hazards regression models for vitrectomy (S5121)

| **Vitrectomy** | Sex | Low FLI | Intermediate FLI | High FLI |
| --- | --- | --- | --- | --- |
| Yes | Men | 1 | NA | NA |
|  | Women | 1 | NA | NA |
| No | Men | 1 | 1.049 (0.982–1.121) | 1.110 (1.028–1.199) |
|  | Women | 1 | 1.053 (0.998–1.110) | 1.183 (1.099–1.273) |

FLI: fatty liver index

Adjusted for age, smoking status, drinking status, physical activity, economic status, body mass index, systolic blood pressure, total cholesterol, preoperative ocular characteristics, diabetes mellitus, dyslipidaemia, and hypertension.

**C**. Multivariable Cox proportional hazards regression models for Uveitis (H22, H20.88, H20.9, H44.1)

| **Uveitis** | Sex | Low FLI | Intermediate FLI | High FLI |
| --- | --- | --- | --- | --- |
| Yes | Men | 1 | 1.047 (0.242–4.521) | 2.364 (0.479–11.675) |
|  | Women | 1 | 0.296 (0.045–1.941) | 6.939 (0.781–61.647) |
| No | Men | 1 | 1.050 (0.982–1.122) | 1.109 (1.027–1.198) |
|  | Women | 1 | 1.054 (0.999–1.112) | 1.184 (1.100–1.274) |

FLI: fatty liver index

Adjusted for age, smoking status, drinking status, physical activity, economic status, body mass index, systolic blood pressure, total cholesterol, preoperative ocular characteristics, diabetes mellitus, dyslipidaemia, and hypertension.

**D**. Multivariable Cox proportional hazards regression models for Scleritis (H15)

| **Scleritis** | Sex | Low FLI | Intermediate FLI | High FLI |
| --- | --- | --- | --- | --- |
| Yes | Men | 1 | 1.019 (0.653–1.592) | 1.126 (0.687–1.861) |
|  | Women | 1 | 1.190 (0.855–1.658) | 1.300 (0.806–2.098) |
| No | Men | 1 | 1.051 (0.983–1.124) | 1.110 (1.027–1.200) |
|  | Women | 1 | 1.050 (0.995–1.108) | 1.180 (1.096–1.271) |

FLI: fatty liver index

Adjusted for age, smoking status, drinking status, physical activity, economic status, body mass index, systolic blood pressure, total cholesterol, preoperative ocular characteristics, diabetes mellitus, dyslipidaemia, and hypertension.
