# Supplementary material for: Association Between Fatty Liver Index and Incidence of Cataract Surgery in Individuals Aged 50 Years and Older Based on the Korean National Health Insurance Service-Health Screening Cohort (NHIS-HEALS) Data: Longitudinal Retrospective Cohort Study
Source: JMIR Public Health Surveill. 2024 Nov 14;10:e57168. doi: 10.2196/57168 (PMC11581417; doi:10.2196/57168)
Supplement: Multimedia Appendix 3 [file publichealth-v10-e57168-s003.docx]

Appendix 3.

Additional information about effect of age on cataract surgery incidence

**Table 2**. Multivariable Cox proportional hazards regression models for all-cataract surgery

|  | **Men** |  |  | **Women** |  |  |
| --- | --- | --- | --- | --- | --- | --- |
| **FLI** | **Low** | **Intermediate** | **High** | **Low** | **Intermediate** | **High** |
| Model 0 | 1 | 0.974 (0.916–1.036) ^c^ | 0.994 (0.938–1.053) ^d^ | 1 | 1.292 (1.233–1.355)* | 1.639 (1.558–1.725)* |
| Model 1 | 1 | 1.102 (1.036–1.172)* | 1.246 (1.175–1.320)* | 1 | 1.092 (1.042–1.145)* | 1.288 (1.224–1.356)* |
| Model 2 | 1 | 1.095 (1.029–1.164)* | 1.223 (1.154–1.297)* | 1 | 1.092 (1.042–1.145)* | 1.289 (1.224–1.357)* |
| Model 3 | 1 | 1.050 (0.983–1.123)^a^ | 1.111 (1.028–1.199)* | 1 | 1.054 (0.999–1.111)^b^ | 1.184 (1.101–1.274)* |

FLI: fatty liver index

* : P-value <0.001, ^a^ : P-value = 0.147, ^b^ : P-value = 0.055, ^c^ : P-value = 0.402, ^d^ : P-value = 0.830

Model 0: unadjusted

Model 1: adjusted for age

Model 2: adjusted for smoking status, drinking status, physical activity, and economic status in addition to the variable of Model 1

Model 3: adjusted for body mass index, systolic blood pressure, total cholesterol, preoperative ocular characteristics, diabetes mellitus, dyslipidaemia, and hypertension in addition to variables of Model 2

**Table 3**. Multivariable Cox proportional hazards regression models for senile-cataract surgery

|  | **Men** |  |  | **Women** |  |  |
| --- | --- | --- | --- | --- | --- | --- |
| **FLI** | **Low** | **Intermediate** | **High** | **Low** | **Intermediate** | **High** |
| Model 0 | 1 | 0.979 (0.918–1.043)^g^ | 1.007 (0.949–1.068)^h^ | 1 | 1.142 (1.088–1.199)* | 1.368 (1.2985–1.441)* |
| Model 1 | 1 | 1.038 (0.975–1.107)^a^ | 1.130 (1.065–1.200)* | 1 | 1.012 (0.964–1.062)^d^ | 1.180 (1.120–1.244)* |
| Model 2 | 1 | 1.034 (0.970–1.101)^b^ | 1.109 (1.044–1.178)* | 1 | 1.011 (0.964–1.062)^e^ | 1.177 (1.117–1.241)* |
| Model 3 | 1 | 1.034 (0.966–1.108)^c^ | 1.106 (1.022–1.197)* | 1 | 1.003 (0.950–1.060)^f^ | 1.147 (1.065–1.237)* |

FLI: fatty liver index

* : P-value <0.05, ^a^ : P-value = 0.244, ^b^ : P-value = 0.309, ^c^ : P-value = 0.336, ^d^: P-value = 0.627, ^e^: P-value = 0.646, ^f^: P-value = 0.913,

^g^: P-value = 0.502, ^h^: P-value = 0.826

Model 0: unadjusted

Model 1: adjusted for age

Model 2: adjusted for smoking status, drinking status, physical activity, and economic status in addition to the variable of Model 1

Model 3: adjusted for body mass index, systolic blood pressure, total cholesterol, preoperative ocular characteristics, diabetes mellitus, dyslipidaemia, and hypertension in addition to variables of Model 2
