# Supplementary material for: Association Between Fatty Liver Index and Incidence of Cataract Surgery in Individuals Aged 50 Years and Older Based on the Korean National Health Insurance Service-Health Screening Cohort (NHIS-HEALS) Data: Longitudinal Retrospective Cohort Study
Source: JMIR Public Health Surveill. 2024 Nov 14;10:e57168. doi: 10.2196/57168 (PMC11581417; doi:10.2196/57168)
Supplement: Multimedia Appendix 4 [file publichealth-v10-e57168-s004.docx]

Appendix 4.

Additional information about effect of age on cataract surgery incidence

Multivariable Cox proportional hazards regression models around the age of 60

| **Age** | Sex | Low FLI | Intermediate FLI | High FLI |
| --- | --- | --- | --- | --- |
| Age ≥ 60 | Men | 1 | 1.033 (0.950–1.122) | 1.088 (0.987–1.201) |
|  | Women | 1 | 1.132 (1.057–1.213) | 1.315 (1.201–1.441) |
| Age < 60 | Men | 1 | 1.029 (0.921–1.150) | 1.082 (0.955–1.225) |
|  | Women | 1 | 0.998 (0.917–1.087) | 1.112 (0.984–1.257) |

FLI: fatty liver index

Adjusted for age, smoking status, drinking status, physical activity, economic status, body mass index, systolic blood pressure, total cholesterol, preoperative ocular characteristics, diabetes mellitus, dyslipidaemia, and hypertension.
